# Benefit-finding experiences of liver transplant recipients who underwent perioperative extracorporeal membrane oxygenation treatment: a qualitative research

**DOI:** 10.3389/fpsyg.2025.1500575

**Published:** 2025-05-30

**Authors:** Zhiying Lei, Yuanyuan Mo, Huiqiao Huang, Lina Huang, Jiehui Zhou, Jianhui Dong, Juhua Zeng, Lu Liu, Xuyong Sun

**Affiliations:** ^1^Department of Organ Transplantation, The Second Affiliated Hospital of Guangxi Medical University, Nanning, China; ^2^Department of Nursing, The Second Affiliated Hospital of Guangxi Medical University, Nanning, China

**Keywords:** liver transplant recipients, ECMO, benefit-finding, perioperative, qualitative research

## Abstract

**Objective:**

This study aimed to examine the benefit-finding experiences of liver transplant recipients who undergo perioperative extracorporeal membrane oxygenation (ECMO) treatments in order to provide targeted nursing care and promote mental health among these patients.

**Methods:**

This study employed the phenomenological research approach within the framework of qualitative research. The determination of the sample size was predicated on achieving information saturation. Between June 2023 and March 2024, eight liver transplant recipients who underwent perioperative ECMO treatment at a qualified liver transplantation hospital in Guangxi were purposively selected for semi-structured interviews. In line with the cognitive adaptation theory, this study employed template analysis as the analytical approach, wherein each word in the text content was meticulously examined and categorized into their respective structures based on the theory's three frameworks. Throughout the analysis process, the researchers continuously refined and adjusted abstract content placed within templates while exploring the potential emergence of new themes.

**Results:**

The study involved a total of eight participants, comprising seven male and one female patient, aged between 43 and 68 years. Among the eight patients, seven had grade 3 or higher coronary artery disease, and one had severe arrhythmia, all of which were attributed to heart conditions necessitating ECMO support during liver transplantation. The ECMO types used were exclusively VA, with auxiliary durations varying between 5 h and 9.5 h. Three overarching themes and seven corresponding sub-themes were extracted: (1) the search for meaning, including a strong desire to survive, recognize the meaning of therapy; (2) gaining a sense of mastery, including a sense of control over one's body and psychological; (3) self-enhancement, including perceiving social support, strengthening self-management, and affirming self-worth.

**Conclusions:**

Liver transplant recipients who undergo preoperative ECMO treatment can have benefit-finding experiences. This study indicates that medical professionals should not only focus on timely and accurate treatment but also prioritize addressing patients' psychological needs while caring for critically ill individuals. Clinical medical staff can guide patients in engaging in positive psychological construction, exploring and providing effective social support resources, fostering patients' self-health management, and enhancing the level and patients' ability of benefit-finding by strengthening knowledge, education, and establishing psychological mutual assistance platforms after liver transplantation.

## 1 Introduction

Liver disease accounts for 2 million deaths and is responsible for 4% of all deaths (1 outof every 25 deaths worldwide); ~2/3 of all liver related deaths occur in men (Devarbhavi et al., 2023). Chronic liver disease and its associated complications have caused significant mortality, morbidity, and economic burden worldwide (Younossi et al., 2023). Liver transplantation involves the surgical replacement of a diseased liver and implantation of a healthy liver to save a patient's life. Since Starzl et al. (1963) first introduced liver transplantation technology into clinical practice in 1963, after more than half a century of advancement, along with the refinement of surgical techniques and the development and application of novel immunosuppressants, the global liver transplantation field has achieved remarkable progress. Liver transplantation is the only effective treatment method for end-stage liver diseases (Ling et al., 2022). The first clinical liver transplant in China was performed in 1977; however, Chinese liver transplantation technology has developed rapidly through continuous research and innovation over the past four decades (Zhao et al., 2025). To date, the cumulative total of liver transplant procedures performed in China has approached 40,000 (Hong et al., 2025). The postoperative survival rates of liver transplant recipients in China from 2015 to 2021 demonstrated a steady improvement. Specifically, the 1-year, 3-year, and 5-year cumulative survival rates for recipients of cadaveric liver transplants were 83.7%, 74.5%, and 68.9%, respectively, while those for recipients of living-donor liver transplants were 92.4%, 89.3%, and 88.2%, respectively (Dou et al., 2024).

However, managing liver transplantation in critically ill patients remains a challenge, especially in transplant recipients with severe cardiopulmonary dysfunction. Compared to other non-cardiac surgeries, liver transplant recipients exhibited a 4fold greater risk of cardiac arrest and ventricular arrhythmias (Sanaiha et al., 2018). Patients with liver cirrhosis classified as Child-Pugh B and C have an exceptionally high risk of requiring surgical valve repair prior to liver transplantation, with a 30-day case fatality rate exceeding 30% (Koshy et al., 2021). During the perioperative period, some patients with end-stage liver disease require extracorporeal membrane oxygenation (ECMO) therapy due to severe cardiopulmonary dysfunction or other comorbidities (Chen et al., 2023; Altshuler et al., 2023). ECMO, a technique that supports respiratory and circulatory functions through a special artificial heart-lung bypass, replaces the functions of the heart and lungs by oxygenating the blood and reintroducing it into the arteries (VA-ECMO) or the venous system (VV-ECMO) to maintain oxygenation and blood supply to organs and tissues (Tyler et al., 2024). ECMO can provide separate gas exchange to support acute respiratory failure (VV-ECMO) or provide combined gas exchange and hemodynamic support to treat complex cardiopulmonary failure (VA-ECMO or VV-ECMO). Patients who undergo LT often have cardiopulmonary dysfunction preoperatively; hence, the use of large-volume fluid resuscitation during surgery and postoperative immunosuppressive agents may exacerbate post-transplant cardiopulmonary complications. Additionally, patients with liver failure and sepsis have distributive hypovolemia, requiring improvement in cardiac output to reverse the anaerobic metabolic process; thus ECMO, is an important cardiopulmonary support technique (Vinogradsky et al., 2025). Moreover, studies have shown that ECMO is effective as a salvage therapy for critically ill patients who require liver transplantation (Patel et al., 2024; Qiu and Hilmi, 2024).

The perioperative period of liver transplantation necessitates the implementation of ECMO treatment for specific patients with critical end-stage liver disease, thereby contributing to their survival. Studies have demonstrated that up to 41% of ECMO-treated survivors experience post-traumatic stress disorder (PTSD) symptoms, which is double the prevalence observed in general intensive care survivors (Luo et al., 2019). Consequently, it is imperative to closely monitor the psychological wellbeing of these individuals. While previous studies (Zhao et al., 2019; Lv et al., 2023; Yan et al., 2024) have focused on the general psychological status of liver transplant recipients. However, there is a lack of research on the psychological status of liver transplant patients who undergo ECMO treatment during the perioperative period. Given the limited research on the psychological processes of ECMO-treated patients and the unclear mechanisms underlying their psychological distress. Therefore, drawing up on positive psychology, this study aims to explore the beneficial discovery experience of this particular group.

Benefit discovery refers to the perception of positive changes experienced by individuals in response to adverse life events. It involves a process of positive cognitive adaptation and behavioral coping (Linley and Joseph, 2004; Affleck and Tennen, 1996). Cognitive Adaptation Theory (CAT), proposed by Taylor (Taylor et al., 1984) in 1983, serves as one of the theoretical foundations for understanding benefit discovery. According to this theory, individuals accumulate social psychological resources during the positive psychological process of coping with extreme stressful events or situations in order to adapt to environmental pressures. Foreign studies have demonstrated that CAT has universal applicability in explaining patients' positive cognitive adaptation. Therefore, based on the CAT theory, we analyzed the experience of benefit discovery among liver transplant recipients who received ECMO treatment during the perioperative period from three perspectives using the following themes: exploration of significance, gaining a sense of control, and self-enhancement. This study's findings may facilitate targeted nursing care and promote the mental health of these patients.

## 2 Methods

### 2.1 Study design

The study was a qualitative research. Data were collected from June 2023 to March 2024.

### 2.2 Participants

A purposive sampling method was employed to select liver transplant recipients who underwent perioperative ECMO treatment as the research subjects in the Organ Transplantation Department of the Second Affiliated Hospital of Guangxi Medical University. The determination of the sample size was predicated on achieving information saturation.

The study's inclusion criteria were as follows: ① Patients who have undergone liver transplantation and received perioperative ECMO therapy; ② Age ≥18 years; ③ Within 3 months post liver transplantation; ④ Perioperative survival; and ⑤ Proficiency in effective communication. The exclusion criteria included the following: ① unconscious or comatose patients and ② Patients with concurrent organ transplantation.

### 2.3 Interview outline

The data collection method employed in this study was interviews. To enhance the quality and scientific rigor of the interviews, an interview team was established. This team consisted of eight members, all of whom were experienced doctors and nurses specializing in organ transplantation and critical care. Among them, two held doctoral degrees, two held master's degrees, and they had an average of 12 years of professional experience.

The interview outline was developed for this study. The design of the interview outline is centered around the research question and adheres to the following six principles: (1) Language expression should be concise; (2) Questions must be specific and targeted; (3) The number of questions should be appropriate, ideally ranging from 6 to 8; (4) Open-ended questions should be used while avoiding leading or suggestive phrasing; (5) Interview questions should maintain structural consistency; (6) There should be logical coherence among the questions, with each being interconnected in either a parallel or progressive relationship (Liu J. et al., 2024).

After consulting relevant literature both domestically and internationally, guided by cognitive adaptation theory and taking into account the specific characteristics of liver transplantation and ECMO treatment, we developed an initial draft of the interview outline. This process referenced the Benefit Finding Scale (Benefit Finding Scale, BFS; Yan et al., 2023). After drafting the initial version of the interview outline, we submitted it to the experts for review and subsequently revised it based on their feedback. Three patients were then selected for pilot interviews. Issues identified during these pilot interviews were addressed through organized team meetings, which facilitated discussions, further analysis, and refinement of the outline. Ultimately, the finalized version of the interview outline for this study was established. The interview primarily consisted of the following open-ended questions: ① Share the psychological changes you have experienced following the illness; ② Have you heard about liver transplantation before? Share your psychological thoughts about receiving liver transplantation; ③ Have you heard about ECMO before? Share your psychological thoughts about receiving ECMO treatment; ④ Share the positive changes you have perceived; ⑤ and Share your future plans.

### 2.4 Data collection

The interviewers selected for this study consisted of one physician and one nurse from the organ transplantation department. These professionals were primarily responsible for diagnosing, treating, and providing nursing care to patients during their hospital stay. Due to their extensive interaction with patients, they possessed a comprehensive understanding of the patients' conditions and had successfully established a strong rapport based on trust.

The interview was conducted in a serene and comfortable conference room. Prior to the interview, the interviewees were given a clear explanation of the research's purpose, format, and timing while ensuring their privacy was safeguarded throughout the entire process. After obtaining informed consent from the interviewees, all discussions were recorded. During the interview, the interviewer refrained from imposing personal opinions in order to allow the interviewees to fully express their individual perspectives. Additionally, prompt responses were encouraged while attentively observing and speculating on their psychological activities and behavioral motives. Whenever the interviewee deviated from answering specific questions, timely redirection toward relevant topics was done using skillful questioning techniques employed to gather necessary details. Finally, the interviews are limited to approximately 30 min.

### 2.5 Data analysis

The data was transcribed into electronic text within 24 h of its acquisition to avoid difficulties or vague recollections that may arise from prolonged time. This also ensured accurate preservation of information and scene records. In line with the cognitive adaptation theory, this study employed template analysis (Zheng and Yu, 2018) as the analytical approach, wherein each word in the text content was meticulously examined and categorized into their respective structures based on the theory's three frameworks. Throughout the analysis process, the researchers continuously refined and adjusted abstract content placed within templates while exploring the potential emergence of new themes.

### 2.6 Quality control

The researchers received comprehensive training in qualitative research and possess extensive experience and proficiency in conducting interviews. Throughout the interview process, the researchers adeptly adjusted the order and methodology based on the interview outline guidance and feedback from the participants. Additionally, valuable questions were pursued without any form of inducement or interference, while respecting the language preferences of interviewees. Following each interview, two researchers independently transcribe and meticulously proofread recorded content to ensure accurate data collection and transcription alignment. Subsequently, both researchers collaboratively analyzed all interview texts, engaging in thorough discussions within the research team to establish a final thematic framework that enhances logical reasoning while upholding data authenticity and credibility.

## 3 Results

**Figure 1:**
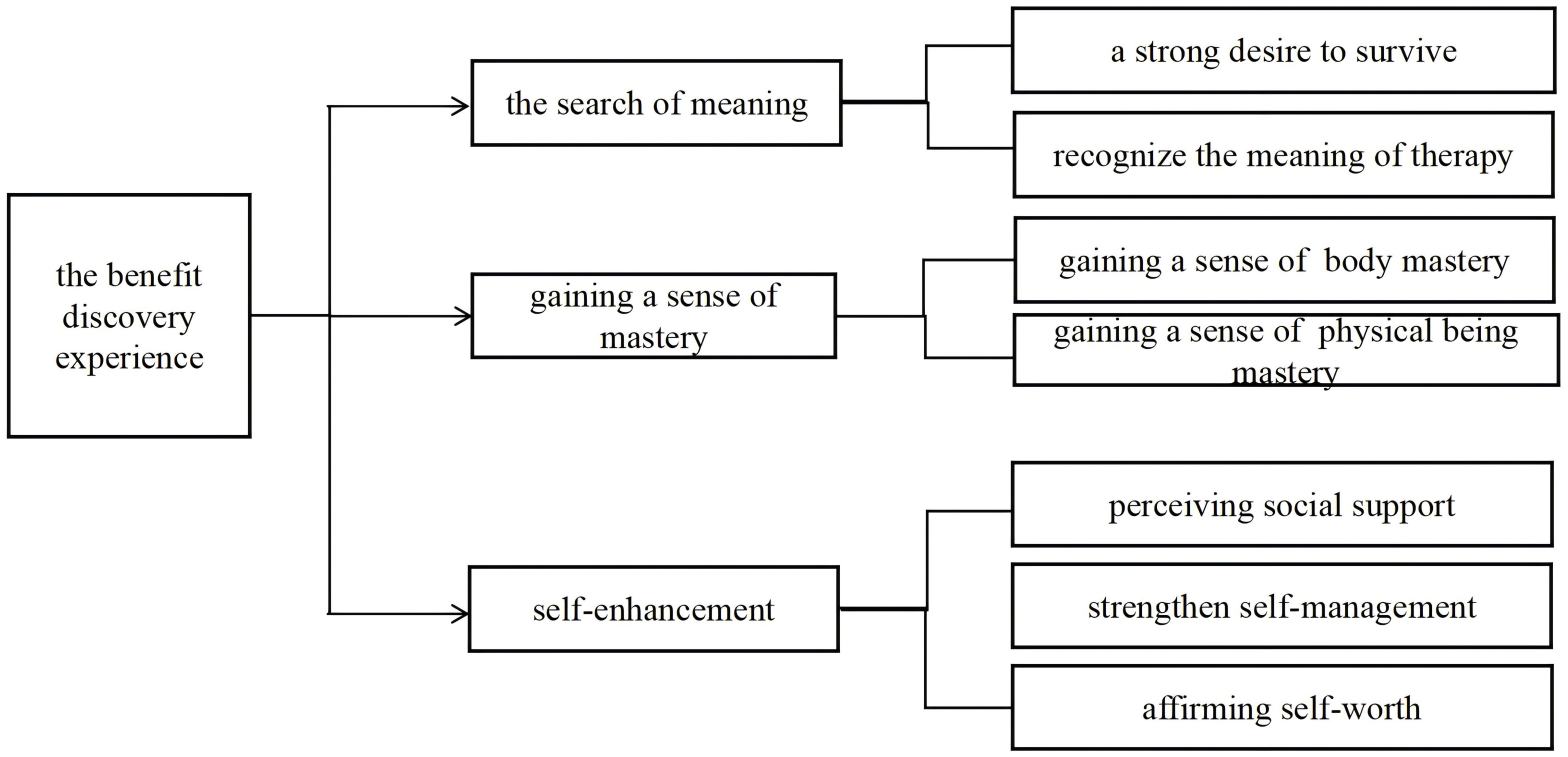
Themes and subthemes. The three themes and seven sub-themes that emerged from the data; each is presented in detail, with participant quotes to illustrate each theme or sum-theme.

The study involved a total of eight participants, comprising seven male and one female patient, aged between 43 and 68 years. All participants were married. The initial diagnoses of the eight patients were as follows: two cases of liver tumors (post-comprehensive treatment), one case of hepatocellular carcinoma, one case of acute-on-chronic liver failure, one case of decompensated liver cirrhosis, one case of primary hepatic carcinoma, and two cases of alcoholic cirrhosis in the decompensation stage. Among the eight patients, seven had grade 3 or higher coronary artery disease, and one had severe arrhythmia, all of which were attributed to heart conditions necessitating ECMO support during liver transplantation. This was done to prevent cardiac arrest that could result from excessive hemodynamic fluctuations during the procedure. The ECMO was initiated following general anesthesia and could be discontinued at the conclusion of the surgery. The ECMO types used were exclusively VA, with auxiliary durations varying between 5 h and 9.5 h. Table 1 presents the general information of the interviewees. Three overarching themes and seven corresponding sub-themes were extracted, as shown in Figure 1.

**Table 1:** General information of the interviewees (*n* = 8).

**ID**	**Sex**	**Age (year)**	**Marital statuses**	**Education**	**Principal diagnosis**	**Date of liver transplantation**	**Date of ECMO treatment start**	**Type of ECMO**	**ECMO duration (hours)**
P1	Male	61	Married	Junior college or below	Liver tumor (after comprehensive treatment)	2023-6-1	2023-6-1	VA	9.5
P2	Male	60	Married	Junior college or below	hepatocellular carcinoma	2023-6-2	2023-6-2	VA	9
P3	Male	68	Married	Junior college or below	Chronic acute liver failure	2023-9-9	2023-9-9	VA	6
P4	Female	55	Married	Undergraduate	decompensated liver cirrhosis	2023-10-14	2023-10-14	VA	7
P5	Male	61	Married	Junior college or below	primary hepatic carcinoma	2023-11-26	2023-11-26	VA	5
P6	Male	67	Married	Junior college or below	Alcoholic cirrhosis decompensation stage	2023-12-19	2023-12-19	VA	8.5
P7	Male	43	Married	Undergraduate	Alcoholic cirrhosis decompensation stage	2023-12-28	2023-12-28	VA	5.5
P8	Male	61	Married	Junior college or below	Liver tumor (after comprehensive treatment)	2024-2-8	2024-2-8	VA	6.5

### 3.1 Topic 1: the search for meaning

After the diagnosis of end-stage liver disease necessitating liver transplantation and ECMO treatment, patients actively sought meaning from their illness and medical interventions, and subsequently constructed a renewed attitude and value toward life.

#### 3.1.1 A strong desire to survive

The respondents unanimously expressed their proactive pursuit of diverse treatments following the diagnosis of end-stage liver disease, demonstrating a profound realization of life's significance and an unwavering determination to survive. P1: “After I planned to have a liver transplant, I queued up in several hospitals in Beijing and Shanghai, but I was still worried about the shortage of liver sources. I could not wait for the operation day. After the patient introduced me to this hospital in Guangxi, I flew from Beijing without hesitation. As long as there was a glimmer of hope, I had to hold on to it.” P5: “I have visited large and small hospitals, and I have received all kinds of prescriptions, just to try what can be done to completely cure liver cirrhosis.”

Most of the respondents expressed prioritizing their life after illness, and placing increased emphasis on their health following liver transplantation. P4: “While I was in a sober state, I told the doctor to prioritize saving my life without considering financial implications.” P7: “I visit the hospital promptly whenever I experience post-surgical discomfort, as I am concerned that a minor issue might escalate into a major complication.”

#### 3.1.2 Recognize the meaning of therapy

The respondents and their families acquired knowledge about liver transplantation through diverse channels and awaited the opportunity for liver transplantation. P4: “Having received insights from my friend's son, who is a medical student, regarding liver transplantation, I conducted extensive online research. After thorough comparison with alternative treatment options, I resolutely opted for liver transplantation.” P8: “As a patient diagnosed with liver cancer, when I was on the verge of losing hope, I discovered the possibility of liver transplantation. At that moment, I clung to this glimmer of hope and eagerly anticipated the day a suitable liver source would become available.”

The patient consented to liver transplantation and ECMO treatment, expressing their endorsement and recommending these interventions to other individuals with end-stage liver disease. P1: “Without receiving a liver transplant, my chances of survival would have been extremely slim.” P2: “I was critically ill, but a liver transplant and ECMO provided me with a renewed lease on life.” P5: “I underwent emergency surgery for hepatic coma, and although I was previously unaware of liver transplantation and ECMO, these two treatments ultimately saved my life. I highly recommend former patients to consider them as well.”

### 3.2 Gaining a sense of mastery

This term refers to the process by which patients, when confronted with events such as illness and treatment, disrupt their initial life trajectory and reconstruct their sense of physical and mental control within an imbalanced environment.

#### 3.2.1 Gaining physical control

The patients developed enhanced physical functioning and a heightened perception of physical mastery. P6: “Prior to the operation, I experienced significant ascites and abdominal distension. Currently, my ascites has completely resolved, resulting in noticeable improvement in my mobility.” P7: “Previously, I had jaundiced skin and eyes along with poor appetite. Following surgery, both my skin and eye color returned to normal, and my appetite has significantly improved.” P8: “Currently, more than 2 months post-operation, an excellent recovery is evident as all my body indicators are within normal ranges. The liver transplant gave me a second chance at life.”

#### 3.2.2 Mental control

From the initial diagnosis of liver disease to the progression toward end-stage liver failure, individuals transitioned from an initial state of denial to eventual acceptance, and acquired a sense of psychological control through effective coping strategies, such as cognitive restructuring and adaptive behaviors. P3: “During our time, vaccination was unavailable. I contracted hepatitis B at a very young age and resigned myself to the possibility of developing cirrhosis in a few decades.” P4: “The onset of my illness was sudden, progressing from liver failure to requiring liver transplantation within just 1 month. Initially, it was psychologically difficult to accept this reality. However, after reviewing the situation multiple times and realizing that this outcome was inevitable, I had to change my mindset and persuade myself to come to terms with it.”

Through cognitive processes, patients can self-adjust their psychological state toward stability as they cope with complications following liver transplantation. P7: “Currently, there are two areas of my wound where fat liquefaction is occurring. According to the doctor's prognosis, it may take one or two months for these areas to fully heal. However, it seems insignificant compared to the overall journey of life and death that I have endured during this process.”

### 3.3 Self-enhancement

The term refers to the phenomenon where patients, in response to distressing events, exhibit varying degrees of shock and selectively attend to positive information pertaining to themselves. They adopt a positive self-perception as a means of safeguarding and enhancing their self-esteem.

#### 3.3.1 Perception of social support

Patients perceive support from various sources, including relatives, friends, medical staff, organ donors, and social networks. P1: “I would like to express my gratitude to my family for their decision regarding the liver transplant and other medical interventions during my coma. I would also like to extend my heartfelt appreciation to the donor and their family for their unwavering commitment.” P2: “The medical professionals in this hospital possess exceptional skills and exhibit a warm demeanor. We appreciate the quality of care provided.” P5: “Despite my family's modest financial situation, they selflessly sold our house to fund my treatment. Their act of sacrifice deeply moved me. Additionally, the substantial reimbursement from medical insurance alleviated a significant portion of the financial burden. I am truly fortunate to have been born into such a supportive family and during an era with advanced healthcare resources.”

The patients also hope to provide support in various ways. P3: “As a recipient of an organ transplant, my family and I have all registered as organ donors to perpetuate this act of love.” P7: “The optimal way to express my gratitude toward my family and organ donors is by prioritizing my health.” P8: “I plan to make annual donations to the Red Cross as a means of contributing to society.”

#### 3.3.2 Enhance health consciousness and bolster self-care capabilities

The patients' understanding of health management was heightened, leading them to actively acquire pertinent information regarding postoperative recovery. P1: “Previously, I did not value my physical wellbeing, but now I am cautious. It is essential for me to appreciate the health that I worked hard to achieve.” P6: “To acquire the relevant knowledge post-surgery, I actively joined multiple patient groups and frequently sought advice from fellow patients within these communities.” P7: “I now engage in daily self-monitoring of my weight and blood pressure to track any changes, ensuring a comprehensive understanding of my health status.”

The patients demonstrated excellent adherence to medical treatment, effectively engaged in health management, and proactively modified detrimental behavioral patterns. P2: “I am currently experiencing a slight deficiency in protein intake. The nurse has provided me with guidance on the types of foods that are rich in protein, which I diligently noted down and would prepare for myself.” P3: “The medical professionals advised against the consumption of alcohol and tobacco. Although it was challenging, I adhered to their advice.” P8: “ Previously, I had no interest in sports; however, I currently engage in daily walks to prioritize my physical wellbeing.”

#### 3.3.3 Enhance one's sense of self-worth

The interviewees validated their own self-worth and cultivated a sense of purpose through altruistic acts and introspection into the significance of their existence. P1: “I will share my transplant narrative with fellow patients, providing valuable insights and guidance to those suffering from end-stage liver disease. My aim is to offer them information and instill hope through my personal experience.” P4: “I aspire to contribute as a volunteer, utilizing my abilities to empower others and bring greater significance to life.” P7: “My child is still in the early stages of development, and being with me will facilitate his optimal growth.”

## 4 Discussion

### 4.1 Liver transplant recipients who undergo preoperative ECMO treatment have a valuable opportunity for benefit discovery

The interviewees had experienced traumatic events, including liver transplant surgery and ECMO treatment, which can elicit beneficial emotions and lead to transformative changes. The findings of various studies indicate that when an individual is confronted with a highly stressful event or situation and fails to fully comprehend the objective facts, it triggers the activation of subjective construction through cognitive adaptation (Taylor et al., 1984; St Fleur et al., 2025; Siefring et al., 2024). During this process of adaptation, the mental functioning of individuals' can recuperate or even surpass their previous level (Choi et al., 2025).

Benefit discovery is not a direct result of liver transplant surgery and ECMO treatment but rather a cognitive adaptation process in which patients derive positive outcomes from adversity. This refers to the experience of responding positively to an unfortunate event. According to Maslow's hierarchy of needs theory (Erdoğan and Mersin, 2025), it is essential to first meet physiological and safety needs. All eight liver transplant recipients who participated in the interviews experienced normalization of their transplanted liver function, alleviation of pre-existing cardiac issues, and significant improvement in overall health compared to their pre-surgical condition. This may be one of the factors that stimulated their experience of beneficial discovery.

### 4.2 Experience of benefits can facilitate the physical and mental recovery of patients

In this study, the respondents exhibited an active response to stressful events such as liver transplantation and ECMO treatment, actively acknowledging the positive impact on individuals and uncovering the transformative changes brought about by challenging or traumatic experiences. This finding aligns with the recognized theory of adaptation proposed by Taylor et al. (1984).

Three overarching themes and seven corresponding sub-themes were extracted: (1) the search for meaning, including a strong desire to survive, recognize the meaning of therapy; (2) gaining a sense of mastery, including a sense of control over one's body and psychological; (3) self-enhancement, including perceiving social support, strengthening self-management, and affirming self-worth. The three overarching themes represent distinct dimensions within the cognitive adaptation process, collectively facilitating the recovery of mental function in liver transplant recipients who undergo perioperative ECMO from both their disease and critical life-support treatment. These three themes constitute a dynamic synergistic process. Simultaneously, there exists a progressive and cyclical relationship among them: meaning exploration serves as the starting point for cognition, a sense of control acts as the foundation for action, and self-improvement represents the ultimate goal. These three dimensions may interact in a cyclical manner (for instance, an enhanced sense of control can further promote the discovery of new meanings).

The process and structure of cognitive adaptation, serving as intermediate regulatory factors, can help individuals to effectively manage stress while fostering positive motivation, enhancing creative output, and attaining physical and mental wellbeing as well as subjective happiness amidst highly stressful circumstances.

This study adopts a qualitative study method, and the results obtained are similar to those obtained by previous studies on burn survivors and lung cancer survivors using quantitative methods (Juan et al., 2025; Zhao et al., 2024). Studies have indicated that experiential knowledge not only alleviates negative emotions in patients but also enhances patient compliance and overall quality of life by promoting positive emotions.

### 4.3 The level of benefit exploration among liver transplant recipients should be enhanced

After undergoing liver transplantation, recipients are required to adhere to long-term immunosuppressant therapy and receive lifelong follow-up care. In China, these patients are included in the chronic disease management program for “anti-rejection therapy after organ transplantation.” Enhancing the quality of life for these individuals is a crucial objective. Research has demonstrated that a higher level of patients' benefit discovery correlates with improved quality of life and overall health (Li et al., 2024; Zhou et al., 2024; Miao et al., 2024; Zhang et al., 2024). The question of how to enhance the level of benefit discovery regarding perioperative ECMO treatment for liver transplant survivors merits careful consideration.

The participants in this study exhibit characteristics of advanced age and lower educational attainment, with a subset relying on medical professionals for their healthcare needs, thereby indicating limited proficiency in self-health management. Therefore, medical staff should assist patients in developing a proactive self-management mindset, adhering to the principle of starting with simplicity, and gradually introducing complexity. This approach would enable patients to comprehend the relevant aspects of their condition and progressively enhance their health literacy and ability for self-care. The utilization of Internet platforms and information tools, such as WeChat and mobile medical mini-programs, simultaneously expands patients' access to knowledge while ensuring a gradual and continuous acquisition of knowledge. Based on the patients' learning ability and specific circumstances, tailored self-management goals should be collaboratively formulated to enhance their confidence in self-health management and their sense of disease control.

In addition, it is crucial to provide patients with multiple and effective social support resources in order to facilitate their reintegration into society. The findings of this study revealed that all participants acknowledged the significance of familial and peer support, compassionate care from healthcare professionals, as well as the protective measures implemented by government medical systems. These external factors were perceived as instrumental in bolstering patients' confidence and motivation toward treatment (Zhao et al., 2024; Liu Y. et al., 2024; Qian et al., 2024). This indicates that medical professionals should not only focus on timely and accurate treatment but also prioritize addressing patients' psychological needs while caring for critically ill individuals. By carefully identifying patients' psychological needs, healthcare professionals can provide timely humanistic care, thereby promoting both the physical and mental wellbeing of patients. Given the lifelong follow-up required after liver transplantation, which incurs substantial costs, it is recommended that medical personnel proactively encourage patients to leverage available social resources while also enlisting the assistance and emotional support of family members, relatives, and friends within their means.

## 5 Conclusions, limitations, and future perspectives

This study employed qualitative research methods to investigate the experience of benefit discovery among patients who undergo simultaneous ECMO treatment and liver transplantation, contributing to a deeper understanding of positive psychology in this context. The findings indicate that this group may experience benefit discovery in major traumatic events, such as severe illness and advanced life-support treatment (ECMO). This study revealed that liver transplant recipients who undergo perioperative ECMO treatment can discover benefits, including three themes of searching for meaning, gaining a sense of control, and self-enhancement. The findings have significant implications for targeted interventions in subsequent stages and can facilitate the physical and mental rehabilitation of patients.

This study indicates that medical professionals should not only focus on timely and accurate treatment but also prioritize addressing patients' psychological needs while caring for critically ill individuals. Clinical medical staff can guide patients in engaging in positive psychological construction, enhancing their self-health ability, exploring and providing effective social support resources, and promoting their physical and mental rehabilitation through strengthened knowledge education and the establishment of psychological mutual assistance platforms after liver transplantation.

According to the different sources of liver transplantation, it can be categorized into living donor liver transplantation and deceased donor liver transplantation. The limitation of this study lies in its inclusion of only deceased donor liver transplantation patients, with a lack of interviews from living donors. In future research, it is recommended to conduct a multicenter study that includes eligible living donor liver transplantation patients as well as underage deceased donor liver transplantation patients. Psychological issues arising from cardiac conditions were not addressed in the development of the interview outline for this study, and this area warrants further investigation in future research.

Furthermore, further exploration is needed regarding the specific manifestations and timing mechanism of patient benefit discovery during their disease-fighting process.

## Data Availability

The raw data supporting the conclusions of this article will be made available by the authors, without undue reservation.
